# Occupational Class Differences in Body Mass Index and Weight Gain in Japan and Finland

**DOI:** 10.2188/jea.JE20130023

**Published:** 2013-11-05

**Authors:** Karri Silventoinen, Takashi Tatsuse, Pekka Martikainen, Ossi Rahkonen, Eero Lahelma, Michikazu Sekine, Tea Lallukka

**Affiliations:** 1 Population Research Unit, Department of Social Research, University of Helsinki, Helsinki, Finland; 2 Department of Welfare Promotion and Epidemiology, University of Toyama, Toyama, Japan; 3 Hjelt Institute, Department of Public Health, University of Helsinki, Helsinki, Finland; 4 Finnish Institute of Occupational Health, Helsinki, Finland

**Keywords:** occupational class, body mass index, weight gain, international comparisons

## Abstract

**Background:**

Occupational class differences in body mass index (BMI) have been systematically reported in developed countries, but the studies have mainly focused on white populations consuming a Westernized diet. We compared occupational class differences in BMI and BMI change in Japan and Finland.

**Methods:**

The baseline surveys were conducted during 1998–1999 among Japanese (*n* = 4080) and during 2000–2002 among Finnish (*n* = 8685) public-sector employees. Follow-up surveys were conducted among those still employed, in 2003 (*n* = 3213) and 2007 (*n* = 7086), respectively. Occupational class and various explanatory factors were surveyed in the baseline questionnaires. Linear regression models were used for data analysis.

**Results:**

BMI was higher at baseline and BMI gain was more rapid in Finland than in Japan. In Finland, baseline BMI was lowest among men and women in the highest occupational class and progressively increased to the lowest occupational class; no gradient was found in Japan (country interaction effect, *P* = 0.020 for men and *P* < 0.0001 for women). Adjustment for confounding factors reflecting work conditions and health behavior increased the occupational class gradient among Finnish men and women, whereas factors related to social life had no effect. No statistically significant difference in BMI gain was found between occupational classes.

**Conclusions:**

The occupational class gradient in BMI was strong among Finnish employees but absent among Japanese employees. This suggests that occupational class inequalities in obesity are not inevitable, even in high-income societies.

## INTRODUCTION

Obesity is a major public health problem worldwide, and its prevalence is likely to remain high in the future.^1^ Obesity is an important risk factor for many chronic diseases, including cardiovascular diseases and type 2 diabetes.^2^ However, in addition to its general effects on population health, obesity substantially contributes to socioeconomic health inequalities in total mortality and the incidences of many chronic diseases.^3^ Two reviews, which together encompassed the findings of 477 studies, concluded that low socioeconomic position was systematically associated with a higher probability of obesity in high-income countries^4^^,^^5^; similar associations were found for weight gain.^6^ In these studies, socioeconomic position was measured using multiple indicators, such as education, income, and occupational position, all of which yielded largely similar results.

The substantial differences in obesity prevalence among populations have led to debate regarding whether some environments increase the risk of obesity. Such environments are referred to as obesogenic and are regarded as typical of many Western societies.^7^ A limitation of previous studies of socioeconomic differences in body mass index (BMI) is that virtually all investigated white populations that consumed a Westernized diet and were experiencing an obesity epidemic, ie, individuals in a putative obesogenic environment. Studies in middle- and low-income countries have yielded more-mixed results on socioeconomic differences in obesity,^4^^,^^5^ but these results may not be comparable to those from high-income countries because of the different role of undernutrition. Two studies of the Japanese population produced mixed results: either no association of BMI with occupational class^8^ or a lower BMI among persons in lower occupational classes.^9^ Thus there are limited data regarding whether an obesogenic environment can modify socioeconomic differences in obesity in a population with a high standard of living.

In this study we assessed the association of occupational class with BMI and change in BMI in Japan and Finland. These 2 countries provide an interesting opportunity for comparative study since they are both industrialized nations with a high standard of living. However, these societies differ from a public health perspective. In the Seven Countries Study, started in 1958, researchers evaluated both the traditional Finnish diet, which is characterized by high consumption of dairy products leading to high intake of saturated fats, and the traditional Japanese diet, characterized by high consumption of vegetables and fish.^10^ The Japanese diet has since changed but is still regarded as healthier than the diets in most Western countries.^11^ Despite an increase in BMI—especially among adult men in Japan^12^—the prevalence of overweight in Japan remains very low as compared with other industrialized countries. In contrast, among Western countries, Finland has one of the highest prevalences of overweight among adults.^1^ More specifically, we analyzed whether occupational class was related to BMI and BMI change during follow-up and if factors related to work, social life, and health behavior explained such differences among Japanese and Finnish public-sector employees.

## METHODS

The Japanese data were derived from the Japanese Civil Servants Study, which enrolled local-government employees in a province on the west coast of Japan.^13^ Data from participants aged 20 to 60 years at baseline were included in the present study. The baseline survey was conducted in 1998–1999, using 4 separate mail questionnaires. Ultimately, we were able to collect information on both BMI and occupational class from 4080 participants (response rate 81%; 30% women). However, the variables used as covariates were collected using different questionnaires; thus, information on covariates was missing for some participants (12%–19%). The follow-up survey was conducted in 2003 among all persons who were employed at the time of the survey. In this study we included only those who had also participated in the baseline survey (*n* = 3213, 30% women). The response rate for the follow-up survey was 79%, and the median duration of follow-up was 4.1 years.

The Finnish data were derived from the Helsinki Health Study, which enrolled employees of the City of Helsinki (the capital of Finland on the south coast of Finland) in 5 age cohorts (40, 45, 50, 55, and 60 years).^14^ The baseline survey was conducted in 2000–2002 (*n* = 8685; 80% women), and the follow-up survey was conducted in 2007; the response rates were 67% and 83%, respectively. The follow-up questionnaire was sent to all participants who participated in the baseline survey. However, we included in this study only those who remained employed at the time of the follow-up survey, to make the data more comparable with the Japanese data. In this cohort (*n* = 7086, 82% women), the median duration of follow-up was 6.5 years. The difference in the proportions of women in these 2 cohorts reflects differences between Japan and Finland in the sex ratio of municipal employees. The median age of participants at baseline was 42 years among men and 40 years among women in Japan and 50 years among men and women in Finland. To test the effect of the larger age variation in the Japanese cohort as compared with the Finnish cohort, we confirmed the main results in datasets restricted to participants aged 40 to 60 years at baseline.

In Japan the height and weight of participants were measured by public health nurses during health examinations at baseline and follow-up. In Finland height and weight were self-reported at baseline and follow-up. BMI was calculated by dividing weight in kilograms by the square of the height in meters (kg/m^2^). Occupational class was classified as manager, professional, clerical employee, and manual worker in both cohorts. This classification scheme is based on a widely used classification of social position that includes upper- and lower-level non-manual workers and skilled and unskilled manual workers. However, this classification was somewhat modified for the present study: skilled and unskilled manual workers were combined because this difference is not clear among public-sector employees. In addition, upper-level non-manual workers were further subdivided into 2 levels because this class is very large in the public sector. Among Japanese there were so few female managers (*n* = 9) and manual workers (*n* = 11) that we combined the former group with the professionals and excluded the latter group from the analyses. To ensure comparability, female Finnish managers were combined with professionals. In Japan, occupational class was self-reported. In Finland, data on occupational class were derived from the personnel registers of employers (78%) or were collected from questionnaires, among those who did not consent to register linkage.

A number of items related to work, social life, and health behavior were included in the baseline questionnaire and were used in this study as confounding or mediating factors. Job control and demands were measured using the Karasek job strain questionnaire and classified into 4 classes (low job strain, passive work, active work, and high job strain).^15^ Working overtime was dichotomized based on whether the respondent normally worked 40 hours or less or 41 hours or more per week. Marital status was classified as married/cohabiting or other. Social relations were ascertained by asking about the number of friends met at least once per month and classified into 5 categories (0, 1–2, 3–5, 6–10, and ≥11). Intensity and duration of physical activity during leisure time and commuting were also determined by analyzing responses to questionnaire items. In Japan, the numbers of hours per week spent engaging in light, medium, and heavy physical activity were surveyed. In Finland, the numbers of hours per week spent participating in the following exercises (or other, equivalent exercises) were surveyed: walking, brisk walking, jogging, and running. On the basis of the intensity and duration of exercise, metabolic equivalent (MET) scores were calculated and dichotomized using the lowest quintile of the distribution, to indicate physical inactivity. Alcohol consumption was surveyed as number of alcohol units consumed (eg, a bottle of beer or glass of *sake* or *shochu* in Japan or wine in Finland) and transformed to grams of pure alcohol per week (1 unit corresponds to 12 grams of alcohol). Alcohol consumption was then categorized into 4 categories, as in other studies that used these data^16^: 0, 1–47, 48–191, and more than 191 grams of pure alcohol per week. For all confounding factors, participants with missing data were classified in a separate category and thus included in the analyses.

We first used baseline BMI as the dependent variable and occupational class as an independent variable. The highest occupational class was used as the reference category (ie, managers for men and professionals for women). The parameter estimates can thus be interpreted as mean difference in BMI units (kg/m^2^) as compared with the reference category. Age was associated with baseline BMI in Japan (*P* = 0.007 for men and *P* < 0.0001 for women) and Finland (*P* < 0.0001 for men and women). After adjustment for the linear effect of age, age-squared was associated with baseline BMI among Japanese men (*P* = 0.003) but not among either Japanese women (*P* = 0.98), Finnish men (*P* = 0.14), or Finnish women (*P* = 0.97). However, to ensure comparability, we adjusted for age and age-squared in all analyses. We then included factors related to work, social life, and health behavior into the model in 3 phases, to analyze whether these factors affected observed relations between occupational class and BMI. After that we conducted similar analyses for BMI change between the baseline and follow-up surveys, using BMI change per 5 years as the dependent variable in the regression models. This was calculated by dividing the difference between BMI at baseline and follow-up by duration of follow-up in years and multiplying the result by 5. Finally, we conducted Cox regression analysis of overweight and obesity during follow-up. The conventional level of significance (*P* < 0.05) was used, and all reported *P*-values are 2-sided. All models were estimated using Stata/SE, version 11.2 (StataCorp, College Station, Texas, USA).

The study of Japanese civil servants was approved by an ad hoc committee of the civil service, comprising the ordinary members of the Safety and Health Committee, labor representatives, and personnel representatives. The Finnish Helsinki Health Study was approved by the Ethics Committee of the Department of Public Health, University of Helsinki and the Ethics Committee of the health authorities of the City of Helsinki. All participants gave written informed consent to participate in the studies.

## RESULTS

Table 1 presents the descriptive statistics for BMI and BMI change per 5 years, by occupational class, in Japan and Finland. Before adjustment for covariates, both mean BMI and BMI change were larger in Finland than in Japan. When we adjusted the results for age and age-squared in the pooled data, mean BMI was 2.82 kg/m^2^ (95% CI, 2.60–3.03) higher among Finnish men and 2.82 kg/m^2^ higher (95% CI, 2.51–3.12) among Finnish women as compared with Japanese men and women, respectively. For mean BMI change per 5 years, the age-adjusted difference between Japan and Finland was 0.32 kg/m^2^ (95% CI, 0.20–0.44) among men and 0.80 kg/m^2^ (95% CI, 0.66–0.93) among women. Using unadjusted BMI values, a gradient in occupational class was seen only for Finnish women, among whom BMI was 1.6 kg/m^2^ lower among professionals as compared with manual workers. For unit change in BMI, no clear gradient in occupational class was seen in Japan or Finland. Only slight differences in BMI were observed between the categories of possible confounding or mediating factors (eTable 1).

**Table 1.** Distribution of participants and mean (SD) BMI and BMI change by occupational class in Japan and Finland

	Japan					Finland				
	
	%	Baseline		BMI change		%	Baseline		BMI change	
		BMI		per 5 years			BMI		per 5 years	
			
		mean	SD	mean	SD		mean	SD	mean	SD
Men										
Managers	8	23.5	2.71	−0.11	1.58	43	25.9	3.53	0.25	1.26
Professionals	47	23.4	2.85	0.29	1.51	19	26.7	4.28	0.21	1.31
Clerical employees	36	23.2	2.93	0.33	1.42	10	26.5	4.32	0.39	1.87
Manual workers	9	23.8	2.73	0.04	1.28	28	26.0	2.97	0.39	1.87
All	100	23.4	2.86	0.27	1.47	100	26.4	3.91	0.31	1.40
		(*n* = 2859)		(*n* = 2264)			(*n* = 1737)		(*n* = 1306)	

Women										
Professionals^a^	70	21.6	2.97	0.13	1.34	46	24.7	4.14	0.54	1.51
Clerical workers	30	21.4	2.78	0.17	1.42	42	25.8	4.50	0.61	1.72
Manual employees	—	—		—		12	26.3	4.80	0.53	1.85
All	100	21.5	2.92	0.14	1.36	100	25.3	4.42	0.57	1.64
		(*n* = 1221)		(*n* = 1221)			(*n* = 6948)		(*n* = 5778)	

^a^Managers and professionals combined.

We continued the analysis by using regression models for baseline BMI (Table 2). Among Finnish men and women, a clear gradient in occupational class was seen, ie, mean BMI progressively increased from the highest to the lowest occupational class. Adjusting for work characteristics (Model 2) slightly widened occupational class differences, which were further increased when indicators of health behavior were included in the model (Model 4). Adjustment for social-life variables had no effect on these estimates (Model 3). No occupational class gradient was seen among the Japanese participants, and differences between occupational classes were not statistically significant. Interaction effects between country and occupational class were statistically significant among men (*P* = 0.015) and women (*P* < 0.0001; female Finnish manual workers were excluded from the analysis) when adjusted for age, age-squared, and their country interaction effects. In datasets restricted to participants aged 40 to 60 years at baseline, the interaction effect between occupational class and country remained statistically significant for baseline BMI among men (*P* = 0.027) and women (*P* = 0.0006).

**Table 2.** Regression coefficients for BMI at baseline by occupational class in Japan and Finland

	Model 1		Model 2		Model 3		Model 4	
				
	β	95% CI	β	95% CI	β	95% CI	β	95% CI
Japanese men (*n* = 2859)								
Managers	ref.		ref.		ref.		ref.	
Professionals	−0.15	−0.59, 0.29	−0.12	−0.57, 0.32	−0.13	−0.57, 0.32	−0.13	−0.57, 0.31
Clerical employees	−0.33	−0.79, 0.14	−0.27	−0.74, 0.20	−0.27	−0.74, 0.21	−0.26	−0.74, 0.21
Manual workers	0.19	−0.34, 0.73	0.29	−0.27, 0.85	0.28	−0.29, 0.84	0.31	−0.26, 0.88
*P*-value for trend	0.005		0.029		0.026		0.034	

Finnish men (*n* = 1737)								
Managers	ref.		ref.		ref.		ref.	
Professionals	0.74	0.24, 1.23	0.77	0.26, 1.28	0.78	0.27, 1.29	0.71	0.20, 1.21
Clerical employees	0.80	0.16, 1.43	0.95	0.29, 1.61	0.95	0.28, 1.62	0.96	0.29, 1.62
Manual workers	1.12	0.67, 1.56	1.28	0.76, 1.79	1.26	0.74, 1.78	1.18	0.66, 1.71
*P*-value for trend	<0.0001		<0.0001		<0.0001		<0.0001	

Japanese women (*n* = 1221)								
Professionals^a^	ref.		ref.		ref.		ref.	
Clerical employees	0.31	−0.03, 0.64	0.29	−0.09, 0.66	0.28	−0.66, 0.10	0.28	−0.66, 0.10
*P*-value for trend	0.075		0.135		0.144		0.144	

Finnish women (*n* = 6948)								
Professionals^a^	ref.		ref.		ref.		ref.	
Clerical employees	1.11	0.89, 1.32	1.20	0.97, 1.44	1.20	0.97, 1.44	1.25	1.02, 1.49
Manual workers	1.58	1.24, 1.91	1.69	1.34, 2.04	1.69	1.34, 2.04	1.80	1.45, 2.15
*P*-value for trend	<0.0001		<0.0001		<0.0001		<0.0001	

Model 1 = age + age-squared; Model 2 = Model 1 + job control and demands + working overtime; Model 3 = Model 2 + marital status + social relations; Model 4 = Model 3 + smoking + alcohol use + physical inactivity.

^a^Managers and professionals combined.

We then conducted corresponding analyses for weight change (Table 3). BMI gain was somewhat higher in the lower occupational categories, but the differences were not statistically significant. The age-adjusted interaction effects for weight gain between country and occupational class were not statistically significant among men (*P* = 0.11) or women (*P* = 0.91).

**Table 3.** Regression coefficients for BMI change by occupational class in Japan and Finland

	Model 1		Model 2		Model 3		Model 4	
			
	β	95% CI	β	95% CI	β	95% CI	β	95% CI
Japanese men (*n* = 2264)								
Managers	ref.		ref.		ref.		ref.	
Professionals	0.20	−0.17, 0.57	0.21	−0.16, 0.58	0.19	−0.18, 0.56	0.19	−0.18, 0.56
Clerical employees	0.22	−0.16, 0.61	0.27	−0.11, 0.65	0.25	−0.13, 0.63	0.25	−0.13, 0.64
Manual workers	0.01	−0.43, 0.44	0.15	−0.30, 0.59	0.16	−0.28, 0.61	0.16	−0.29, 0.61
*P*-value for trend	0.761		0.535		0.434		0.444	

Finnish men (*n* = 1306)								
Managers	ref.		ref.		ref.		ref.	
Professionals	−0.06	−0.26, 0.15	−0.08	−0.29, 0.13	−0.08	−0.29, 0.13	−0.07	−0.28, 0.14
Clerical employees	0.04	−0.22, 0.30	0.02	−0.25, 0.29	−0.01	−0.28, 0.27	−0.03	−0.31, 0.24
Manual workers	0.17	0.02, 0.36	0.11	−0.11, 0.32	0.10	−0.12, 0.31	0.10	−0.13, 0.32
*P*-value for trend	0.079		0.345		0.419		0.450	

Japanese women (*n* = 1221)								
Professionals^a^	ref.		ref.		ref.		ref.	
Clerical employees	−0.10	−0.32, 0.13	−0.14	−0.40, 0.11	−0.13	−0.38, 0.13	−0.11	−0.36, 0.15
*P*-value for trend	0.397		0.257		0.329		0.399	

Finnish women (*n* = 5778)								
Professionals^a^	ref.		ref.		ref.		ref.	
Clerical employees	−0.02	−1.16, 0.12	−0.03	0.17, 0.12	−0.02	−0.16, 0.13	−0.01	−0.15, 0.15
Manual workers	0.05	−0.09, 0.19	0.06	−0.08, 0.20	0.06	−0.08, 0.20	0.07	−0.07, 0.21
*P*-value for trend	0.311		0.319		0.412		0.545	

Model 1 = age + age-squared; Model 2 = Model 1 + job control and demands + working overtime; Model 3 = Model 2 + marital status + social relations; Model 4 = Model 3 + smoking + alcohol use + physical inactivity.

^a^Managers and professionals combined.

Finally, we analyzed overweight and obesity during follow-up (Table 4). Using BMI values at the end of follow-up, the prevalences of overweight/obesity (BMI > 25 kg/m^2^) and obesity (BMI > 30 kg/m^2^) were much higher in Finland (54% and 18%, respectively) than in Japan (23% and 3%). Thus, we used obesity in Finland and overweight/obesity in Japan as the outcome. When we analyzed these outcomes using Cox proportional hazards models, a clear gradient in occupational class was seen in Finland; however, among Japanese no gradient was found for men and overweight/obesity was rarer among female clerical employees than among female professionals. Because different outcomes were used, we did not conduct a formal interaction test, as we did for the analysis of BMI.

**Table 4.** Hazard ratios (HRs) for obesity/overweight (BMI > 25 kg/m^2^) in Japan and obesity (BMI > 30 kg/m^2^) in Finland during follow-up

	Model 1		Model 2		Model 3		Model 4	
			
	HR	95% CI	HR	95% CI	HR	95% CI	HR	95% CI
Japanese men (*n* = 2264)								
Managers	ref.		ref.		ref.		ref.	
Professionals	0.90	0.56, 1.46	0.91	0.56, 1.47	0.91	0.56, 1.48	0.88	0.54, 1.43
Clerical employees	0.79	0.48, 1.30	0.82	0.49, 1.35	0.82	0.50, 1.36	0.80	0.48, 1.33
Manual workers	0.90	0.51, 1.59	0.96	0.53, 1.75	1.00	0.55, 1.83	0.93	0.51, 1.70
*P*-value for trend	0.328		0.544		0.613		0.543	

Finnish men (*n* = 1306)								
Managers	ref.		ref.		ref.		ref.	
Professionals	1.47	1.04, 2.08	1.48	1.04, 2.12	1.50	1.05, 2.15	1.39	0.97, 2.00
Clerical employees	1.49	0.98, 2.26	1.61	1.04, 2.49	1.58	1.02, 2.46	1.57	1.00, 2.45
Manual workers	1.59	1.16, 2.17	1.57	1.09, 2.25	1.48	1.03, 2.13	1.38	0.95, 2.00
*P*-value for trend	0.003		0.012		0.031		0.078	

Japanese women (*n* = 1221)								
Professionals^a^	ref.		ref.		ref.		ref.	
Clerical employees	0.50	0.27, 0.92	0.46	0.23, 0.91	0.49	0.24, 0.97	0.44	0.22, 0.90
*P*-value for trend	0.026		0.025		0.042		0.025	

Finnish women (*n* = 5778)								
Professionals^a^	ref.		ref.		ref.		ref.	
Clerical employees	1.45	1.27, 1.66	1.50	1.29, 1.73	1.49	1.29, 1.72	1.47	1.26, 1.70
Manual workers	1.81	1.51, 2.18	1.90	1.56, 2.31	1.96	1.61, 2.39	1.90	1.55, 2.32
*P*-value for trend	<0.0001		<0.0001		<0.0001		<0.0001	

Abbreviation: BMI, body mass index.

Model 1 = age + age squared; Model 2 = Model 1 + job control and demands + working overtime; Model 3 = Model 2 + marital status + social relations; Model 4 = Model 3 + smoking + alcohol use + physical inactivity.

^a^Managers and professionals combined.

## DISCUSSION

We found clear occupational class differences in BMI and the rate of obesity in Finland but not in Japan. These results are consistent with those of previous studies, which showed clear socioeconomic gradients in obesity in Finland^17^ and more-mixed results in Japan. A previous study of Japanese civil servants found an occupational class pattern in waist-to-hip ratio but not in BMI among men and women,^8^ whereas a study of Japanese male employees found that waist-to-hip ratio and BMI were more favorable in lower social positions.^9^ The absence of a clear occupational class gradient in Japan is noteworthy because socioeconomic differences in BMI have been found in many other high-income countries.^4^^,^^5^ A likely reason for this difference is that the Japanese environment is less obesogenic than the Finnish environment.^1^ This hypothesis is supported by the findings of the present study: both mean BMI and BMI gain were higher in Finland than in Japan. However, it has proven difficult to identify the relevant characteristics of an obesogenic environment. Factors such as easy access to fast-food restaurants and limited possibilities for physical exercise are suggested characteristics of an obesogenic environment, but evidence linking such characteristics with obesity development remains limited.^7^^,^^18^ Further we found that adjusting the results for covariates related to work environment and health behaviors increased rather than decreased BMI differences between occupational classes in Finland. This suggests that the occupational class patterns of mean BMI in Japan and Finland are due to macrosocial factors rather than individual or workplace-related factors.

A likely proximate factor for the observed discrepancies between Japan and Finland in mean BMI, BMI change, and occupational class gradient in BMI is differences in diet. The Japanese diet has traditionally been very healthy, with high consumption of vegetables, soy protein, and fish.^10^ During the last several decades, the Japanese diet has changed toward the Westernized diet, eg, greater consumption of meat and dairy products, which were rare in the traditional Japanese diet. However, the general nutritional status of the Japanese population is still healthier than that in many Western countries.^11^ Socioeconomic differences in nutrition have been found in Northern European populations—in particular, consumption of fruits and vegetables was found to be systematically higher among those in higher social classes.^19^ There is also evidence that manual workers are more conservative in their diet than persons in higher social classes.^20^ This may have led to healthier nutrition among manual workers in Japan and unhealthy nutrition in Finland. In Finland, for example, the traditional diet in the lower social classes includes butter, milk, and meat, whereas more vegetables and fruits are consumed in higher social classes.^21^ Unfortunately we had no data on diet in the Japanese cohort to evaluate these possibilities directly. However, in a previous study we found that occupational class gradient was steeper for physical inactivity among Finns, as compared with Japanese, suggesting possible differences between these countries in the social patterning of health behavior.^22^ In the present study, however, adjustment for physical inactivity did not explain the BMI gradient among Finns.

For BMI change we found only limited evidence of an occupational class gradient, which was previously reported in many other high-income populations.^6^ Even when there was evidence of a greater increase in BMI in lower occupational classes among both Finnish and Japanese employees, the differences were not statistically significant. The lack of statistically significant associations could be due to the relatively short duration of follow-up and/or the smaller sample of Finnish men. It is possible that a larger sample or longer duration of follow-up would have shown greater BMI gains among lower occupational positions.

In addition to dietary differences, other differences between the present study populations should be considered in explaining the discrepancies in the occupational class gradient in BMI. Although Japan and Finland are very different societies, the macro-socioeconomic contexts of our study populations have many similarities. In the year 2000 the gross domestic product per capita was 25 958 US$ in Japan and 25 674 US$ in Finland,^23^ and the infant mortality rate was 3.50 in Finland and 3.30 in Japan per 1000 live births,^24^ which confirms that both societies provide a high level of human welfare. It is also noteworthy that public-sector employees have secure employment in both countries.

However, there are also differences between Japan and Finland. In Japan, advancement at work has traditionally been more closely associated with age than in Finland. The participation of young mothers in the Japanese labor force is low,^25^ whereas there are only small differences between men and women in labor force participation in Finland.^26^ Further, the role of governmental organizations is strong in Japanese society, and public-sector governmental jobs have traditionally been highly regarded.^25^ In contrast, the large public sector in Finland has created a long-term financial burden that has led to a generally lower salary level in the public sector as compared with the private sector. Nevertheless, public-sector employees have a more secure work environment.^27^ Thus, selection of public-sector jobs may differ in these 2 societies. There could also be differences between the 2 countries in macro-level factors that influence occupational positions—factors that are not directly associated with hierarchical social structures.

To determine whether the occupational hierarchy for public-sector employees is comparable in Japan and Finland, we conducted sensitivity analyses using height as a proxy indicator of childhood living conditions (eTable 2), since clear socioeconomic differences in height have been systematically reported from traditional societies to modern, industrialized nations.^28^ We found evidence for a difference in women: the height difference between professionals and clerical workers was much lower in Japan than in Finland (country interaction effect, *P* < 0.0001). This suggests that female clerical workers are socioeconomically more heterogeneous in Japan than in Finland. However, among men we found no differences between Japan and Finland in height gradients by occupational class (country interaction effect, *P* < 0.99). This suggests that, at least among men, occupational classifications reflect true hierarchical stratifications in our study populations.

Our data have both strengths and limitations. The main strength is that we have data on BMI at baseline and follow-up from the populations of 2 high-income countries that substantially differ in obesogenic environmental characteristics. We also have data on occupational class and a number of confounding factors, which were collected using the same questionnaire or closely related wording. A limitation of our study is that data on BMI were self-reported in Finland. The lower reliability of self-reporting measures may have decreased observed differences between occupational classes in Finland, although it is unlikely to be the reason for our main result, ie, the presence of occupational class BMI differences in Finland but not in Japan. It is also noteworthy that our study participants are public-sector employees. Thus our results should be generalized mainly to employees with secure economic conditions; the associations may differ in more-marginalized groups. Further, we lack data on other measures of social position, such as education or income. However, in Japan and Finland, income and education are closely related to the social hierarchy of public-sector employees. Finally, our classification included only 4 occupational classes for men and fewer classes for women. A more detailed occupational classification would have allowed for a more comprehensive analysis of occupational class gradient.

In conclusion, a clear occupational class gradient in BMI was present in Finland (which has a more obesogenic environment, with a high mean BMI and rapid weight gain) but not in Japan (which has a less obesogenic environment, with low mean BMI and slow weight gain). A less obesogenic environment is itself a worthy target, but our results suggest that such an environment could also lead to reduced socioeconomic differences in BMI and thus a more equal distribution of health in society.

## ONLINE ONLY MATERIALS

eTable 1. Distributions of participants and mean BMI by background characteristics in Japan and Finland.

eTable 2. Regression coefficients of height by occupational class in Japan and Finland.
