# Supplementary material for: Occupational Class Differences in Body Mass Index and Weight Gain in Japan and Finland
Source: J Epidemiol. 2013 Nov 5;23(6):443–50. doi: 10.2188/jea.JE20130023 (PMC3834282; doi:10.2188/jea.JE20130023)
Supplement: eTable 1. — Distributions of participants and mean BMI by background characteristics in Japan and Finland. [file je-23-443-s001.pdf]

eTable 1. Distributions of Participants and Mean Body Mass Index by Background Characteristics in Japan and Finland

	**Japan**				**Finland**			
	**Men**		**Women**		**Men**		**Women**	
	**%**	**BMI mean**	**%**	**BMI mean**	**%**	**BMI mean**	**%**	**BMI mean**
Job strain								
Low job strain	28	23.3	26	21.5	26	26.3	25	25.2
Passive work	24	23.6	24	21.7	30	26.4	27	25.5
Active work	23	23.3	27	21.3	25	26.3	25	25.1
High job strain	13	23.1	11	21.7	18	26.9	21	25.6
Missing	12	23.6	12	21.7	1	26.8	2	25.6
Working overtime								
No (≤40 h/week)	32	23.4	28	21.8	77	26.4	85	25.3
Yes (>40 h/week)	49	23.3	54	21.4	21	26.4	13	25.3
Missing	19	23.6	18	21.5	2	27.8	2	25.7
Marital status								
Married or cohabiting	71	23.1	61	20.6	77	26.5	67	25.4
Others	17	23.4	27	21.9	23	26.4	33	25.3
Missing	12	23.4	12	21.5	0	-	0	-
Number of friends met at least once per month								
0	18	23.2	18	21.5	6	25.8	5	25.2
1-2	29	23.5	31	21.5	32	26.5	37	25.4
3-5	23	23.2	26	21.6	35	26.6	37	25.4
6-10	7	23.4	5	21.6	14	26.1	12	25.2
≥11	6	23.5	3	21.7	8	26.5	5	25.2
Missing	17	23.6	17	21.5	5	26.3	4	25.3
Current smoker								
No	50	23.4	79	21.6	72	26.4	77	25.4
Yes	36	23.3	5	21.9	27	26.3	23	25.2
Missing	14	23.5	16	21.2	1	26.9	1	26.0
Physical inactivity								
No	64	23.4	69	21.5	78	26.1	79	25.0
Yes	21	23.3	15	21.9	21	27.7	20	26.7
Missing	15	23.5	16	21.2	1	27.8	1	26.8
Alcohol consumption								
0 g per week	14	23.4	39	21.6	5	26.6	8	26.1
1-47 g per week	13	23.3	25	21.6	37	26.3	63	25.4
48-191 g per week	32	23.3	16	21.5	40	26.5	25	24.9
>191 g per week	25	23.4	2	21.6	17	26.5	3	26.0
Missing	16	23.5	18	21.1	1	27.0	1	25.3
	(N=1221)		(N=1221)		(N=6948)		(N=5778)	
