# Supplementary material for: Occupational Class Differences in Body Mass Index and Weight Gain in Japan and Finland
Source: J Epidemiol. 2013 Nov 5;23(6):443–50. doi: 10.2188/jea.JE20130023 (PMC3834282; doi:10.2188/jea.JE20130023)
Supplement: eTable 2. — Regression coefficients of height by occupational class in Japan and Finland. [file je-23-443-s002.pdf]

eTable 2. Regression Coefficients for Height by Occupational Class in Japan and Finland[a]

	Japanese men		Finnish men		Japanese women[b]		Finnish women[b]	
	**β**	**95% CI**	**β**	**95% CI**	**β**	**95% CI**	**β**	**95% CI**
Managers	ref.		ref.		NA		NA	
Professionals	-0.79	-0.65, -0.61	-0.98	-1.79, -0.17	ref.		ref.	
Clerical employees	-1.16	-2.07, -0.26	-1.33	-2.37, -0.30	-0.13	-0.75, 0.49	-1.40	-1.68, -1.12
Manual workers	-1.57	-2.61, -0.53	-1.66	-2.38, -0.93	NA		-1.42	-1.85, -0.99
p-value for trend	0.002		<0.0001		0.646		<0.0001	
	(N=2859)		(N=1737)		(N=1221)		(N=6948)	

[a]Adjusted age and age-squared
[b]Managers and professionals combined
